# Local Translation in Axons: When Membraneless RNP Granules Meet Membrane-Bound Organelles

**DOI:** 10.3389/fmolb.2019.00129

**Published:** 2019-11-22

**Authors:** Kavya Vinayan Pushpalatha, Florence Besse

**Affiliations:** Université Côte d'Azur, CNRS, Inserm, Institut de Biology Valrose, Nice, France

**Keywords:** RNA transport, local translation, RNP granules, axon, vesicular trafficking, mitochondria

## Abstract

Eukaryotic cell compartmentalization relies on long-known membrane-delimited organelles, as well as on more recently discovered membraneless macromolecular condensates. How these two types of organelles interact to regulate cellular functions is still largely unclear. In this review, we highlight how membraneless ribonucleoprotein (RNP) organelles, enriched in RNAs and associated regulatory proteins, cooperate with membrane-bound organelles for tight spatio-temporal control of gene expression in the axons of neuronal cells. Specifically, we present recent evidence that motile membrane-bound organelles are used as vehicles by RNP cargoes, promoting the long-range transport of mRNA molecules to distal axons. As demonstrated by recent work, membrane-bound organelles also promote local protein synthesis, by serving as platforms for the local translation of mRNAs recruited to their outer surface. Furthermore, dynamic and specific association between RNP cargoes and membrane-bound organelles is mediated by bi-partite adapter molecules that interact with both types of organelles selectively, in a regulated-manner. Maintaining such a dynamic interplay is critical, as alterations in this process are linked to neurodegenerative diseases. Together, emerging studies thus point to the coordination of membrane-bound and membraneless organelles as an organizing principle underlying local cellular responses.

## Introduction

Neurons are highly polarized cells that establish long-distance contacts with numerous other cells by extending cellular processes specialized in information transfer, processing and storage. During nervous system development, neurons in particular extend growing axons that navigate toward specific targets and branch in response to chemical and mechanical cues. Axonal processes then mature into presynaptic terminals that are actively maintained in response to neurotrophic factors and locally remodeled upon neuronal activity. Thus, both immature and mature axons must dynamically adjust their molecular content to respond rapidly to localized extracellular cues. Local translation of mRNAs targeted to axonal compartments has proven to be a very efficient means employed by neuronal cells to regulate their axonal proteome with high spatio-temporal resolution (Jung et al., 2012; Sahoo et al., 2018; Holt et al., 2019). Indeed, recent *in vitro* and *in vivo* transcriptome-wide studies have revealed that up to hundreds of transcripts are found in axons and translated in response to specific cues (Zivraj et al., 2010; Gumy et al., 2011; Shigeoka et al., 2016; Cagnetta et al., 2018; Poulopoulos et al., 2019). Furthermore, functionally relevant changes in the axonal translatome are observed during nervous system maturation, upon switching from axonal elongation to neurotransmission (Shigeoka et al., 2016). Both specific targeting of mRNAs and tight translational regulation are controlled by RNA binding proteins (RBPs) that recognize distinct sets of transcripts and assemble with their targets into macromolecular ribonucleoprotein (RNP) assemblies termed RNP granules (Muller-McNicoll and Neugebauer, 2013; De Graeve and Besse, 2018; Gallagher and Ramos, 2018; Formicola et al., 2019). These granules are actively transported along axons and contribute to translational control dually, on one hand by participating to the repression of their associated mRNAs during transport, and on the other hand by fuelling local protein synthesis upon cue-induced remodeling (De Graeve and Besse, 2018; Formicola et al., 2019). As revealed by recent *in vitro* and *in vivo* work, neuronal RNP granules result from a self-assembly process that generates phase-separated condensates selectively concentrating RNA and protein molecules (Murakami et al., 2015; Patel et al., 2015; Gopal et al., 2017; Shin and Brangwynne, 2017; Tsang et al., 2019). While this discovery nicely explains the dynamic behavior of these membraneless organelles, it does not shed light onto how RNP granules are hooked to the transport machinery for long-distance transport, or how they are linked to the translational machinery.

In this review, we first present recent work describing the material properties of RNP condensates. We then discuss recent evidence suggesting that RNP condensates tightly interact with membrane-bound organelles undergoing active, motor-driven motion for their transport to axons. Tight connections between membraneless RNP granules and axonally-localized membrane-bound organelles are also crucial for translation, as both mitochondria and endosomes were shown to serve as platforms supporting local protein synthesis. Understanding how these connections are regulated is key, and we highlight here the major role played by adapter molecules that bridge the two types of organelles specifically, in response to local signals. In the last part of this review, we present a model whereby targeting of RNP granules to distinct membrane-bound organelles or sub-cellular compartments may lead to stimuli-specific translation activation patterns. Finally, evidence linking altered interactions between RNP granule and membrane-bound organelles with the progression of neurodegenerative diseases is discussed.

## Neuronal RNP Granules Are Membraneless Phase-Separated Organelles

Cellular and biochemical studies have defined neuronal RNP granules as macromolecular entities enriched in RNA and associated RNA binding proteins, and detected as punctate structures by light microscopy (De Graeve and Besse, 2018; Formicola et al., 2019). Characterization of RNP granule content, on one hand, revealed that neuronal RNP granules are not all identical, but rather contain heterogeneous sets of regulatory proteins and target RNAs (De Graeve and Besse, 2018). For example, a minimal overlap was observed in both the protein and the RNA content of RNP granules purified from rat brain using two established RBP markers: Staufen2 and Barentsz (Fritzsche et al., 2013; Heraud-Farlow et al., 2013). Furthermore, differences in granule composition were observed when comparing dendritically- and axonally-localized FMRP-positive granules (Christie et al., 2009). Ultra-structural analyses, on the other hand, demonstrated that RNP granules are not bound by a membrane, defining them as *bona fide* membraneless organelles (Knowles et al., 1996; Krichevsky and Kosik, 2001; Elvira et al., 2006; El Fatimy et al., 2016). If neuronal RNP granules are not delimited by a membrane, how are their constituent molecules then assembling into coherent and delimited entities? Extensive recent work performed in cells and in reconstituted systems has demonstrated that RNP granules in fact behave as liquid-like condensates that form through liquid-liquid phase separation, i.e., by demixing of their components from the cytoplasm (Weber and Brangwynne, 2012; Alberti, 2017; Banani et al., 2017; Mittag and Parker, 2018; Van Treeck and Parker, 2018). Such a self-assembly mechanism relies on the establishment of dense and dynamic networks of RNA-RNA, RNA-protein and protein-protein interactions (Mittag and Parker, 2018; Van Treeck and Parker, 2018). It involves multivalent molecular interactions mediated by repeated domains as well as low-complexity domains that are prone to interact with both RNA and protein and frequently found in neuronal RBPs (Formicola et al., 2019; Franzmann and Alberti, 2019). Consistent with surface tension dictating their morphology, neuronal RNP granules are spherical at rest and deform under the shear stress induced by fast axonal transport (Gopal et al., 2017; Andrusiak et al., 2019). Furthermore, combining high resolution imaging with FRAP experiments revealed that neuronal RNP granules behave as droplets, undergoing fusion with characteristic relaxation times together with rapid internal rearrangements and constant exchange with the surrounding cytoplasm (Cougot et al., 2008; Park et al., 2014; El Fatimy et al., 2016; Gopal et al., 2017; Andrusiak et al., 2019).

A remarkable feature of phase-separated organelles is their capacity to rapidly and reversibly disassemble, or modulate their dynamic properties and composition in response to changes in the phase behavior of their constituent molecules (Banani et al., 2017). In the axons of cultured neurons, for example, TDP-43-containing granules with different properties are observed: while rather static granules with a low turnover rate are observed proximally, highly dynamic granules, strongly dependent on weak hydrophobic interactions, are observed more distally (Gopal et al., 2017). Although the origin of such differences is still unclear, they likely reflect subcellular heterogeneities in the concentrations of granule components, cations, or biological hydrotropes along neuronal processes (Buxbaum et al., 2014; Patel et al., 2017; Onuchic et al., 2019). More acute changes in granule properties are also observed in response to external stimuli. In *C. elegans* mechanosensory neurons, for example, axotomy induces within minutes an increase in the number of TIAR-2-containing axonal granules together with a change in their material properties manifested by a reduction in granule fusion events and circularity (Andrusiak et al., 2019). Point mutations preventing these changes inhibit the function of TIAR-2 in axon regeneration, highlighting the functional importance of controlling phase behavior. Understanding the nature and precise impact of the molecular determinants modulating phase separation has been the subject of intensive research, and it has become clear that modifications of both proteins and RNAs play a very important role in this process. By altering charge or steric properties, post-translational modifications (PTMs) of RNA binding proteins, including phosphorylation, SUMOylation or methylation, were indeed shown to positively or negatively modulate *cis-* and *trans-*interactions, thus altering phase separation and molecule partitioning in reconstituted systems (Hofweber and Dormann, 2019). In neuronal cells, preventing PTMs of granule-associated proteins was shown to alter granule component oligomerization, as well as granule dynamics, nucleation and/or growth (Majumdar et al., 2012; Khayachi et al., 2018; Qamar et al., 2018; Andrusiak et al., 2019; Ford et al., 2019). Such changes in granule properties were associated with impaired axonal translation (Qamar et al., 2018), regenerative capacities (Andrusiak et al., 2019), synaptic properties (Khayachi et al., 2018), or long-term memory (Majumdar et al., 2012; White-Grindley et al., 2014), highlighting that PTMs of neuronal RBPs are essential for the tight regulation of RNP granule function. More recently, chemical modification of RNA molecules, in particular m6A methylation, was also shown to regulate phase behavior *in vitro* and to impact on the recruitment of RNP components in cells (Ries et al., 2019), although evidence for a role of m6A in the assembly and regulation of constitutive neuronal RNP granules is still lacking. Together, these studies have provided a conceptual framework explaining the dynamic regulatory properties of membraneless RNP organelles. To date, a few studies have started investigating how phase behavior impacts on the translation repressor function of RNP granule components (Khan et al., 2015; Kim et al., 2019; Tsang et al., 2019). However, we still lack a precise understanding of how individual activities might be coordinated in the context of these macromolecular complexes. Furthermore, how such dynamic assemblies connect to the transport machinery and travel over long distances along axons remains unclear.

## Membrane-Bound Organelles as Vehicles for Axonal Transport of RNAs

**Figure 1:**
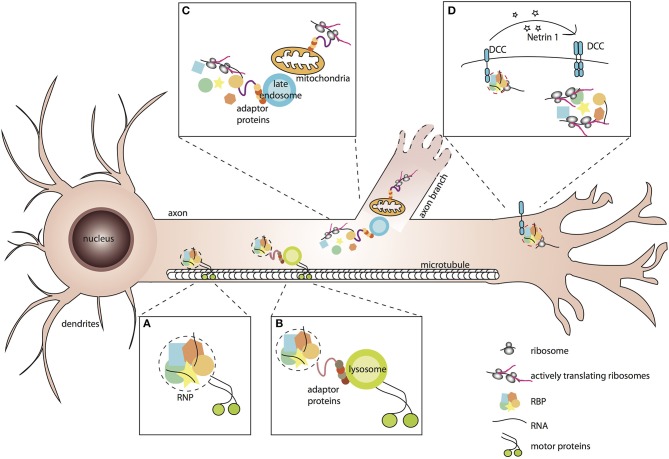
Association of RNP granules to membrane-bound organelles or receptor complexes elicits spatio-temporal responses to environmental stimuli. **(A,B)** RNP granules are membraneless condensates of RNA binding proteins and RNAs that associate with motor proteins for their transport along microtubules. Association with motors may be direct **(A)**, or mediated by tethering to membrane-bound organelles such as lysosomes **(B)**. **(C)** Tight association of RNP granules with late endosomes and mitochondria enables local translation of mitochondrial RNAs and other mRNAs necessary for neurite branching. **(D)** Binding of ligands to their specific receptors triggers the release of RNP complexes and local activation of mRNA translation.

*In vitro* and *ex vivo* live-imaging of fluorescently-tagged mRNAs or their associated RBPs has revealed that RNP assemblies are transported to distal axons through active, bi-directional motion characterized by the presence of both anterograde and retrograde processive events interspaced by long stationary phases (Knowles et al., 1996; Tiruchinapalli et al., 2003; Leung et al., 2006, 2018; Nalavadi et al., 2012; Alami et al., 2014; Medioni et al., 2014; Gopal et al., 2017; Wong et al., 2017; De Graeve and Besse, 2018; Turner-Bridger et al., 2018; Vijayakumar et al., 2019). Long-range transport of RNP granules along the axon shaft relies on the integrity of the microtubule cytoskeleton (Knowles et al., 1996; Medioni et al., 2014; Leung et al., 2018) and likely requires the combined activity of kinesin and dynein motors, although direct evidence is still scarce (Das et al., 2019). How are molecular motors recruited to RNP assemblies for their transport to axons? Physical interactions between neuronal RBPs and molecular motors have been described (Figure 1A; Kanai et al., 2004; Davidovic et al., 2007; Dictenberg et al., 2008; Bianco et al., 2010; Urbanska et al., 2017), suggesting that RBPs may engage motor proteins through direct or adaptor-mediated binding. Recent lines of evidence, however, have challenged this classical view and proposed that RNP cargoes may hitchhike on motile membrane-bound organelles for their subcellular trafficking (Jansen et al., 2014; Salogiannis and Reck-Peterson, 2017).

Intimate connections between localizing mRNAs and ER-related endomembranes have long been described in non-neuronal cells (e.g., vertebrate and invertebrate oocytes, yeast), where ER tubules were shown to promote the targeting of mRNAs encoding membrane and secreted proteins, possibly facilitating their local translation (Trautwein et al., 2004; Cohen, 2005; Schmid et al., 2006). In motor neurons, evidence for axonal co-trafficking of golgi-derived coat protein I (COPI) vesicles and the RNP chaperone SMN, together with the demonstration that COPI subunits physically and functionally interact with axonally-localized mRNAs, had also suggested that RNP trafficking may be facilitated by membrane-bound components (Bi et al., 2007; Peter et al., 2011; Todd et al., 2013).

Direct evidence that membrane-bound organelles undergoing bi-directional, microtubule-dependent motion may serve as vehicles for the transport or mRNA molecules arose more recently, first through elegant work performed in the filamentous fungus *Ustilago maydis*. In this model, Kinesin and dynein-dependent co-transport of Rab5a-positive endosomes and RNP components was observed along the elongating hyphal processes (Baumann et al., 2012, 2014). Furthermore, molecularly uncoupling mRNAs from endosomes prevented mRNA localization without interfering with endosome shuttling, demonstrating that RNP assemblies indeed behave as cargoes and endosomes as vehicles (Baumann et al., 2014; Pohlmann et al., 2015). Together, this work supported an emerging model in which endosomes are not purely dedicated to the sorting or recycling of internalized components, but also serve as versatile multipurpose platforms that recruit and localize signaling molecules (Gould and Lippincott-Schwartz, 2009). Strikingly, extensive colocalization was also recently observed between axonal mRNAs and Rab5-positive early endosomes or Rab7-positive late endosomes in vertebrate Retinal Ganglion neurons (Konopacki et al., 2016; Cioni et al., 2019). As shown by expression of dominant negative versions of Rab5 and 7, however, endosomes appear to be dispensable for the transport of RNP granules in this system (Cioni et al., 2019). Alternative membrane-bound organelles actively transported along axonal microtubules (Farias et al., 2017) may however be used as vehicles in neuronal cells. In a recent study, indeed, Ward and colleagues proposed that RNP granules may hitch a ride on lysosomes for their long-distance transport to axons (Figure 1B; Liao et al., 2019), based on the following lines of evidence: (i) most motile RNP granules co-localized with LAMP1-positive lysosomes, (ii) a tight association of the two organelles was observed by correlative light-electron microscopy, suggestive of a docking mechanism, and (iii) inhibition of motor-dependent lysosomal movement blocked RNP granule transport. Arguing against an indirect effect of lysosomal trafficking impairment, specific disruption of RNP granule hitchhiking on lysosomes reduced the number of RNP granules actively trafficking along axons *in vitro* and *in vivo*. Whether these lysosomal vesicles correspond to mature degradative lysosomes, or rather to the less acidic lysosome-related vesicles recently shown to mediate the transport of presynaptic components (Vukoja et al., 2018), remains to be addressed.

An outstanding question arising from these discoveries is how the tethering of phase-separated RNP condensates on membrane-bound organelles is molecularly achieved. Work performed in *Saccharomyces cerevisiae* first demonstrated that the RNA binding protein She2p possesses lipid-binding properties and specifically recognized membrane structures with a high curvature typical of tubular ER, suggesting that RBPs can directly and specifically bridge the two organelles (Genz et al., 2013). Association between membraneless and membrane-bound organelles may also be mediated by bipartite adapter proteins containing both a membrane binding domain and an RNP association domain. In *Ustilago*, for example, the adapter molecule Upa1 directly couples RNP and endosomes by binding directly to endosomes, through its C-terminal FYVE domains, and to the main RBP involved in mRNA transport, through its PALM2 domains (Pohlmann et al., 2015). Notably, mutating either the FYVE domains or the PALM2 domain of Upa1 prevented association of mRNAs with endosomes and transport, without affecting general endosome functions. Similarly, Annexin A11 was recently proposed to act as an adaptor between RNP granules and lysosomes in mammalian neurons. Annexin A11, indeed, was identified as both a lysosome and an RNP granule interactor by proximity labeling proteomics (Markmiller et al., 2018; Liao et al., 2019). Furthermore, it contains both an N-terminal low-complexity domain mediating phase separation into granule-like droplets *in vitro* and incorporation into stress-induced RNP granules in cells, and C-terminal Annexin domains that bind membranes containing PI(3,5)P_2_ lysosomal lipids (Liao et al., 2019). Remarkably, downregulating Annexin A11 drastically reduced the number of axonal RNP granules trafficking on lysosomes without altering axonal lysosome transport itself. Altered hitchhiking of RNP granules was associated with a decreased accumulation of β*-actin* mRNA in distal axons, indicating the functional relevance of this process in axonal mRNA localization.

Interestingly, docking of RNP assemblies on shuttling membrane-bound organelles is very dynamic, as frequent on and off-loading events were observed by live-imaging in different systems (Higuchi et al., 2014; Cioni et al., 2019; Liao et al., 2019). To date, how the docking process is regulated physiologically largely remains unclear, although work on the Annexin A11 protein has revealed that its interaction with lysosomes is both calcium- and phospholipid-sensitive (Liao et al., 2019). As measured using a FRET sensor monitoring the association between Annexin A11 and the lysosomal protein LAMP1, indeed, chelating free cytoplasmic Ca^2+^, or inhibiting the formation of PI(3,5)P_2_, decreased Annexin A11/lysosome interaction. Such regulatory mechanisms thus provide neurons with flexible means to regulate with high spatial and temporal resolution the trafficking of RNP assemblies.

## Membrane-Bound Organelles as Platforms for Local Translation

For proteins to be produced in axons, mRNA localization must be tightly coupled to translation. Although the capacity of axons to support local translation has been debated over years, combinations of metabolic labeling and proteomic studies have unambiguously demonstrated that both cytosolic and transmembrane proteins can be translated in distal axons (Sahoo et al., 2018; Holt et al., 2019). How the translation machinery is trafficked to axons is still an open question, but the detection of both ribosomal proteins and translation factors in Mass-Spectrometry analyses of RNP granule content has suggested that it may at least partly be co-transported with neuronal RNP granules (Kanai et al., 2004; Elvira et al., 2006; El Fatimy et al., 2016). Importantly, recent studies demonstrating the importance of organelle-coupled translation completed this view (Bethune et al., 2019), indicating that membrane-bound organelles including mitochondria and endosomes may serve as sites for the local translation of a significant fraction of axonal mRNAs.

Nuclear-encoded mitochondrial RNAs (mtRNAs) have long and reproducibly been identified in transcriptomic analyses of axonally localized mRNAs (Taylor et al., 2009; Andreassi et al., 2010; Gumy et al., 2011; Aschrafi et al., 2016), and were more recently shown to be translated in axons (Yoon et al., 2012; Shigeoka et al., 2016; Cagnetta et al., 2018). These observations, together with the discovery that mtRNAs are targeted and translated at the mitochondrial surface (Lesnik et al., 2015), suggest a speculative model whereby mtRNAs might hitchhike on mitochondria for their transport and get translated on axonally localized mitochondria. Consistent with such a model, mitochondria are dynamically transported to axons and enriched distally (Smith and Gallo, 2018). Second, hundreds of nuclear-encoded mtRNAs were shown to accumulate at the mitochondrial outer membrane, a fraction of them being targeted via 3′UTR-located *cis*-regulatory sequences (Marc et al., 2002; Sylvestre et al., 2003; Fazal et al., 2019). Furthermore, as demonstrated for the axonally-localized *cytochrome c oxidase IV* mRNA, the distal region of the transcript's 3′UTR is both required for mitochondrial targeting and for axon localization (Aschrafi et al., 2010). Third, proximity-specific ribosome profiling analysis revealed that hundreds of transcripts encoding mitochondrial proteins are translated at the vicinity of mitochondria, a discovery consistent with the identification of translating ribosomes at mitochondrial outer membranes (Zhang et al., 2016; Gold et al., 2017). Notably, mitochondria were also shown to be required more indirectly, through mitochondrial respiration, for the local translation of axonal mRNAs such as β*-actin, Arp2*, or *cortactin*, as well as for protein synthesis-dependent axon branching (Spillane et al., 2013). During this process, preferential association of mitochondria with sites of active axonal translation was observed, further suggesting a tight coupling of energy supply to RNA translation.

A tight coupling was also observed between mitochondria and late endosomes (Figure 1C), other membrane-bound organelles recently proposed to behave as hot spots for intra-axonal protein synthesis (Cioni et al., 2019). As demonstrated by high resolution imaging of *Xenopus* Retinal Ganglion Cell axons, both RNP components and ribosomes were frequently found in close proximity to Rab7a-positive late endosomes. Furthermore, translation of axonal mRNAs was detected on endosomes, corroborating previous work suggesting endosome-sited translation in heterologous systems (Baumann et al., 2014; Higuchi et al., 2014). Finally, inhibiting late endosomes through downregulation of Rab7 activity, or pharmacological blockage of late endosome maturation, decreased the translation of axonal *laminB2* mRNAs as well as global axonal translation, indicating that late endosomes significantly contribute to local translation (Cioni et al., 2019). Intriguingly, while extensive coupling of RNP granules to early endosomes was also observed in axons, inhibiting early endosome function did not impair axonal translation, indicating the existence of yet unknown specificity mechanism(s). Another important open question concerns the nature and properties of the molecular linker(s) tethering both mRNAs and the translation machinery to endosomes. Identifying such linker(s) will be key to perform more targeted manipulation and should open the door to a mechanistic and functional understanding of endosome-sited translation regulation.

## Subcellular Compartmentalization as a Means to Generate Specific Translational Patterns?

During development, local translation is required for cue-induced axon outgrowth, branching, as well as for the chemotropic response of growth cones to guidance molecules (Campbell and Holt, 2001; Wu et al., 2005; Hengst et al., 2009; Jung et al., 2012; Medioni et al., 2012; Spillane et al., 2012; Wong et al., 2017). Strikingly, seminal studies performed in cultured Retinal Ganglion Cell axons revealed that Netrin-1-induced axon turning is associated with the translation of β*-actin*, while Slit2-induced growth cone collapse is associated with the translation of *cofilin* (Leung et al., 2006; Piper et al., 2006; Lin and Holt, 2008). These results suggested first that different cues trigger translation of different mRNAs, and second that attractive and repulsive cues may stimulate the translation of proteins with opposite functions in the assembly/disassembly of the F-actin cytoskeleton. By providing a more comprehensive view on the nascent proteomes induced by different cues in somaless retinal axons, recent work performed using highly sensitive sample preparation and metabolic labeling both confirmed and completed this view (Cagnetta et al., 2018). Indeed, while the translation of some axonal mRNAs was commonly activated in response to different cues, cue-specific up- and down-regulation of dozens of nascent proteins was also observed. Furthermore, opposite changes in translation patterns were observed upon switching from repulsive to attractive chemotropic responses, suggesting that guidance molecules induce distinct and functionally relevant proteomic signatures.

These observations raise the question of how specificity of translational patterns is achieved (Besse and Ephrussi, 2008). One way to selectively regulate the translation of subgroups of localized mRNAs is to organize them into so-called post-transcriptional operons, or regulons (Keene, 2007), composed of functionally-related RNAs recognized by specific RNA binding protein(s). By targeting their bound RNAs to specialized organelles or subcellular micro-domains, RBPs may thus favor stimuli-specific spatio-temporal responses. Although a systematic profiling of organelle-associated transcriptomes has not been performed in neuronal cells, distinct RBPs and mRNA populations were found associated with different types of membrane-bound organelles, consistent with such a model (Peter et al., 2011; Todd et al., 2013; Debaisieux et al., 2016; Yarmishyn et al., 2016; Liao et al., 2019). Analysis of mRNAs co-precipitating with COPIa, for example, revealed the presence of more than a thousand of mRNAs, 8 to 10% overlapping with the axonal transcriptome and about the same percentage encoding cytoskeletal components and/or regulators (Todd et al., 2013). Furthermore, RNP complexes with specific protein and RNA signatures were recently found in the immunoprecipitates of different guidance cue receptors in neuronal cells (Koppers et al., 2019). The RBP Staufen 1, for example, was found significantly associated with the Neuropilin receptor Nrp1, whereas hnRNPA2/B1 was found interacting with the Netrin-1 DCC receptor. Interestingly, Nrp1 and DCC also bound distinct subsets of mRNAs, the translation of which was exclusively induced by their specific ligand. As demonstrated in *Xenopus* Retinal Ganglion neurons, translation of β*-catenin* (*ctnnb1*) mRNA was for example activated in response to the DCC ligand Netrin-1, but not in response to Sema3A (Koppers et al., 2019). Translation activation correlated with a release of both RNAs and associated ribosomes from the receptor complex, suggesting (i) that mRNAs tethered to receptors are kept in a translationally repressed state and (ii) that cue-induced release may represent a rapid and direct means to trigger selective translation activation (Figure 1D; Tcherkezian et al., 2010; Koppers et al., 2019). How such release is achieved remains to be understood, but a possibility is that cue-induced signaling triggers the phosphorylation of RBPs associated with transmembrane receptors. Signal-specific phosphorylation of neuronal RBPs has already been documented in different contexts and linked to decreased affinity for target mRNAs (Huang et al., 2002; Huttelmaier et al., 2005; Lee, 2012), consistent with the idea that cue-induced signaling may lead to the release of mRNAs from their local anchor. Whether such a model of regulated association/dissociation holds true for mRNAs associated with other sub-cellular organelles or compartments is unclear, and would be worth investigating in the future.

## Alterations in RNP Granule Transport and Their Membrane-Bound Vehicles Are Linked to Neurodegenerative Diseases

Alterations in axonal mRNA transport and local translation impact various aspects of axon function, including axon survival (Jung et al., 2012; Sahoo et al., 2018). Consistent with this, an increasing number of neurodegenerative disease-causing mutations have been mapped to proteins, either RNA binding or adapter molecules, present in axonal RNP granules (Table 1) (Costa and Willis, 2018; Khalil et al., 2018). Interestingly, functional studies of pathogenic mutations have suggested that they may impair RNP cargo transport by at least two different means: (i) by perturbing the assembly or material properties of RNP condensates, or (ii) by altering the tethering of RNP cargoes to motile membrane-bound organelles.

**Table 1:** List of neurodegenerative disease-associated proteins and mutations mentioned in the main text.

**Disease**	**Protein**	**Category of protein**	**Functional domains**	**Molecular function**	**Alterations in RNP regulation upon mutation**	**Examples of disease-associated mutations in the corresponding domains**
Spinal muscular atrophy (SMA)	SMN	RNP chaperone	Protein-protein interaction domain	Mediates interaction with α-COP (K76 and K82)	nd	nd
	α-COP	Subunit of the COPI vesicle	C-terminal domain	Mediates interaction with SMN (Y1090 residue)	nd	nd
Amyotrophic lateral sclerosis (ALS)/Fronto-temporal dementia (FTD)	TDP-43	RNA binding protein	Low complexity domain	Promotes phase separation	Decreased RNP component exchange rate and axonal transport of RNP granules	G298S, M337V
	FUS	RNA binding protein	Low complexity domain	Promotes phase separation	Decreased RNP component exchange rate, impaired axonal translation	G156E
			NLS domain	Promotes nuclear localization	Decreased RNP component exchange rate, impaired axonal translation	R521C, R521H
	Annexin A11	Adaptor protein	Low-complexity domain	Promotes phase separation	Decreased RNP component exchange rate	D40G
			Annexin domains	Association with lysosomes	Decreased RNP component exchange rate, RNP granule trafficking and axonal mRNA localization	R235Q, R346C
Charcot-Marie-Tooth disease type 2B (CMT2B)	Rab7	Small GTPase	GTP binding and hydrolysis domain	GTPase activity	Decreased endosome sited axonal mRNA translation	L129F, K157N, N161T/I, V162M (in Σ3 and Σ4 motifs)
Huntington's disease (HD)	Htt	Scaffolding protein	na	Binding to molecular motors	Decreased dendritic mRNA transport^*^	na

Domains involved and their molecular functions are summarized. nd, not determined; na, not applicable.

* *Studies performed by downregulating (not mutating) Htt*.

A prominent example of neurodegenerative disease linked to altered axonal RNP cargo transport is amyotrophic lateral sclerosis (ALS), a disease characterized by the loss of upper and lower motor neurons (Taylor et al., 2016). Remarkably, while the vast majority of ALS cases are sporadic, about 10% are familial and largely caused by mutations in genes coding for RNA binding proteins (Zhao et al., 2018). Mutations in two components of axonally-localized RNP granules, FUS/TLS and TDP-43, have been particularly studied and their impact on axonal mRNA transport and translation investigated (Yasuda and Mili, 2016). ALS mutations in FUS were shown to inhibit general intra-axonal protein synthesis in cultures of *Xenopus* retinal neurons (Murakami et al., 2015), as well as in mouse sciatic nerve axons *in vivo* (Lopez-Erauskin et al., 2018). Furthermore, mutant TDP-43 proteins, when expressed in cultured neurons, exhibit defective axonal transport characterized by a decreased anterograde movement and a depletion of TDP-43-containing granules from the distal axonal compartment (Alami et al., 2014; Gopal et al., 2017). This phenotype is accompanied by a defective anterograde trafficking of the TDP-43 target mRNA *Neurofilament-L*, both in cultured cortical neurons and in IPS cell-derived human motor neurons carrying ALS-causing mutations, indicating that disrupting the delivery of mRNAs to axons may underlie axonal degeneration (Alami et al., 2014). How the disease mutations molecularly impair mRNA transport and translation remains to be understood, but mutations found in the low-complexity domains of FUS and TDP-43 were shown to alter granule material properties, promoting a liquid-to-solid phase transition (Johnson et al., 2009; Murakami et al., 2015; Patel et al., 2015). The increased viscosity and aggregated state of RNP granules observed in mutant contexts may then directly impact on granule motility and cue-induced remodeling, or more indirectly affect local RNA homeostasis, by sequestration of essential RNA or protein molecules (Ramaswami et al., 2013; Bowden and Dormann, 2016; Alberti and Dormann, 2019). Further emphasizing the importance of RNP granule homeostasis in the etiology of the disease, numerous ALS-associated mutations were found in the Annexin A11 protein (Table 1; Smith et al., 2017). These mutations, found either in the N-terminal low complexity domain, or in the C-terminal lysosome association domains, were shown to induce a solidification of associated RNP granules (Liao et al., 2019). C-terminal mutations also specifically impair the association of Annexin A11 to lysosomes, and, when expressed in primary cultured neurons or *in vivo* in zebrafish neurons, disrupt both hitchhiking of RNP cargoes to motile lysosomes and targeting of mRNAs to the distal end of axons (Liao et al., 2019). Together, these results illustrate how different ALS mutations converge on factors controlling axonal RNP motility and dynamic interaction with membrane-bound organelles, highlighting the likely involvement of this process in disease pathogenesis or progression.

Another example of axonal RNP-associated protein linked to disease is SMN, whose deficiency causes spinal muscular atrophy (SMA), a neurodegenerative disease characterized by a progressive loss of spinal motor neurons and skeletal muscle atrophy (Fallini et al., 2012; Beattie and Kolb, 2018). SMN is an RNP chaperone molecule shown to interact with several RBPs and to be transported within axonal RNP granules undergoing active bi-directional motion. In the absence of SMN, loss of axonal RNP granules and significant decrease in axonal mRNA levels are observed (Rage et al., 2013; Fallini et al., 2014, 2016; Saal et al., 2014), indicating that defective RNP assembly and subsequent axonal transport defects may at least partially lead to SMA. Interestingly, specifically altering the COPI/SMN interaction impairs the developmental function of SMN in axons (Custer et al., 2013, 2019; Li et al., 2015), suggesting that loosing the tight interactions between axonal RNP granules and membrane-bound organelles might also play a role in disease progression. Further supporting the importance of such interactions, mutations in the late endosome protein Rab7 known to be causally linked to the Charcot-Marie-Tooth disease type 2B (CMT2B), disrupted axonal mRNA translation when expressed in cultured retinal neurons (Cioni et al., 2019). Notably, the above-described examples likely reflect only the tip of the iceberg, as defective regulation of axon ribostasis and membrane-bound organelle trafficking is emerging as a general feature of neurodegenerative diseases, including Alzheimer disease or Huntington disease (De Vos et al., 2008; Ma et al., 2011; Ramaswami et al., 2013; Schreij et al., 2016; Khalil et al., 2018; Lie and Nixon, 2019). The Huntingtin (Htt) protein, in particular, was shown to (i) undergo active, bi-directional transport in axons (Gunawardena et al., 2003) and (ii) to associate and co-traffic with both RNP granule components (Savas et al., 2010; Ma et al., 2011) and membrane-bound organelles such as BDNF vesicles or Rab-positive recycling endosomes (Gauthier et al., 2004; White et al., 2015; Saudou and Humbert, 2016). Inactivating Htt impaired the localization of its associated RNAs and membranous organelles (Table 1; Gauthier et al., 2004; Trushina et al., 2004; White et al., 2015), indicating its functional role in connecting these cargoes to the transport machinery. Although Htt was reported to physically interact with the dynein molecular motor (Li et al., 1998; Gauthier et al., 2004; Caviston et al., 2007), its specific normal and pathological functions in cargo recruitment and hitchhiking remain to be clarified, thus emphasizing the need to better understand the molecular bases of RNP granule/membrane-bound organelle co-trafficking.

## Conclusions and Perspectives

Extensive anterograde and retrograde trafficking of both membrane-bound and membraneless cargoes has long been observed along axons and shown to supply the distal ends of these long processes with a source of membranes, proteins and RNAs. While different types of cargoes have largely been studied independent of each other, recent work summarized in this review has demonstrated the dependency of phase-separated RNP granules on membrane-bound vesicles or organelles, both for long-distance transport of RNP cargoes and for local translation of their associated mRNAs. A number of challenging questions still remain to be addressed regarding the specificity of these interactions. For example, it will be very important to characterize the transcriptomes of the different membrane-bound organelles trafficked to axons and thus understand if and how different RNP complexes are specifically targeted to distinct membrane-bound organelles. Furthermore, identifying the adapter molecules tethering RNP granules to their membrane-bound vehicles or anchors will be key to functionally dissect the role and physiological regulation of this interaction. Transcriptomic and proteomic studies required to address these questions will be possible with the advent of technologies providing both high sensitivity and high spatial resolution (Markmiller et al., 2018; Medioni and Besse, 2018; Fazal et al., 2019). Last, it will be interesting to investigate the contribution of specialized ribosomes to the specific translation activation patterns observed in response to different extracellular cues. As suggested by recent work, indeed, ribosomes of different compositions and functional properties may co-exist and exhibit preferential selectivity for subsets of mRNAs (Shi and Barna, 2015; Segev and Gerst, 2018). In axons, remarkable differences in the stoichiometry of defined ribosomal proteins were found when comparing the composition of ribosomes associated with distinct guidance molecule receptors (Koppers et al., 2019). Furthermore, on-site incorporation of axonally-synthesized ribosomal proteins and subsequent remodeling of ribosomes was observed (Shigeoka et al., 2019), thus opening the door for functional dissection of this additional layer of spatio-temporal regulation.

## Author Contributions

KP and FB contributed to manuscript preparation, read, and approved the submitted version.

### Conflict of Interest

The authors declare that the research was conducted in the absence of any commercial or financial relationships that could be construed as a potential conflict of interest.
